# 

**Published:** 1904-02-20

**Authors:** 


					- / THE HOSPITAL. "The Standard of highest purity."?Lancet. February 20th, 1904.
CADBURY'S u CADBURY S
COCOA 1 MM , COCOA
"A PERFECT FOOD." ?W NO DRUGS OR ALKALIES USED.
No. 908. Vol. XXXY. [fS?5??!] Saturday,
Feb. 20th, 1904.
[Subso^pp0368frms'] PRICE THREEPEHCE.

				

